# Work Stress and Metabolic Syndrome in Police Officers. A Prospective Study

**DOI:** 10.1371/journal.pone.0144318

**Published:** 2015-12-07

**Authors:** Sergio Garbarino, Nicola Magnavita

**Affiliations:** 1 State Police Health Service Department, Ministry of the Interior, Rome, Genoa, Italy; 2 Department of Health Sciences, University of Genoa, Genoa, Italy; 3 Department of Neuroscience, Rehabilitation, Ophthalmology, Genetics and Maternal-Infantile Sciences (DINOGMI), Genoa, Italy; 4 Department of Public Health, Università Cattolica del Sacro Cuore, Roma, Italy; Mathematical Institute, HUNGARY

## Abstract

**Objective:**

The aim of this longitudinal study was to evaluate the association between occupational stress and metabolic syndrome (MetS) in a rapid response police unit.

**Method:**

Work-related stress was continuously monitored during the 5-year period with both the Demand-Control-Support (DCS) and the Effort-Reward Imbalance (ERI) models. Blood pressure, body mass index (BMI), waist circumference, triglycerides, HDL-cholesterol, and fasting blood glucose were measured at baseline in January 2009, and in January 2014. 234 out of 290 police officers (81%) completed the follow-up.

**Results:**

The majority of police officers had high stress levels. At follow-up, police officers in the highest quartile of stress had significantly higher mean levels of triglycerides, and lower levels of HDL-cholesterol than their colleagues in the lowest quartile. Police officers with high stress had an increased adjusted risk of developing MetS (aOR = 2.68; CI95% = 1.08–6.70), and hypertriglyceridemia (aOR = 7.86; CI95 = 1.29–48.04). Demand and Effort were significant predictors of MetS.

**Conclusion:**

Our study supports the hypothesis that work-related stress induces MetS, particularly through its effects on blood lipids. Future longitudinal studies with continuous monitoring of stress levels will definitively confirm this hypothesis.

## Introduction

Metabolic syndrome (MetS) is a cluster of risk factors including dyslipidemia, abdominal adiposity, reduced glucose tolerance and hypertension. MetS is highly prevalent and increasing in most parts of the world [1]. A meta-analysis has shown that MetS increases the risk of myocardial infarction, cardiovascular disease, and cardiovascular disease mortality by twofold [1]. MetS is also associated with the risk of cancer [2], especially breast cancer in women [3] and with cognitive decline in younger people [4].

Psychological stress at work is considered to be a risk factor for MetS. A cross-sectional study of Italian radiologists and radiotherapists showed that chronically distressed individuals had a more than four-fold prevalence of MetS compared to colleagues with lower stress levels [5]. Healthy workers who had experienced minor psychological trauma (injury, violence) at the workplace had a significantly higher frequency of metabolic syndrome components than controls (adjusted OR, 1.8; 95% CI, 1.2–2.6); in particular, male workers had a higher rate of hypertriglyceridemia (OR 2.53 CI95% 1.03–6.22), and female workers a higher rate of hypertension (OR 2.45 CI95% 1.29–4.66) [6]. In cross-sectional studies, job strain was significantly related to heart rate variability [7], fasting glucose [8], dyslipidemia and arterial hypertension [9]. The Whitehall II study showed that chronic stress at work increases the incidence of MetS [10]. In the CARDIA longitudinal study on young adults, men in high-demands/high-control (active) jobs and women in high-demand/low-control (high strain) jobs had a significantly increased risk of incident MetS over 5 years [11]. A recent review concluded that an association between chronic psychosocial stress at work and the development of MetS is generally supported, even if too few longitudinal studies are available to reach a definite conclusion [12]. There is also a fundamental problem concerning the assessment of work-related stress in studies in the literature, since measuring stress and metabolic parameters at baseline and reporting changes over many years can be unsatisfactory, as both metabolic variables and the level of stress may fluctuate over time [12].

Several theoretical models have been used to quantify exposure to job strain. In the occupational field, the two most widely used general models are the Job Demand Control Support (DCS) model by Karasek [13] and the Effort–Reward Imbalance (ERI) model by Siegrist et al. [14]. The first model is based on a self-report questionnaire that measures several aspects of an individual’s perception of work: the first aspect is psychological demand and focuses on psychological stress associated with time pressure, work interruptions, task complexity and execution, unplanned tasks and contradictory demands. The second aspect concerns decision latitude, which measures the amount of control an individual has over his/her own work (autonomy and liberty to choose how to carry out the work) and skill use (the possibility of using one’s qualifications and developing new ones). The ERI model analyses the mismatch between the efforts spent and rewards (whether symbolic, pecuniary, of esteem or recognition) received. In several reviews and meta-analyses, these factors have been related to health outcomes such as physical and mental disorders [15, 16].

Police officers have been found to be at a high risk for MetS and coronary heart diseases [17, 18]. Previous cross-sectional studies showed that MetS–related parameters, such as impaired fasting glucose, and impaired glucose tolerance, are more frequent in police officers than in other workers [19], and changes in biochemical parameters are associated with self-perceived stress levels [20]. The Buffalo Cardio-Metabolic Occupational Police Stress study reported a significant association between stress and MetS among a small sample of female officers [21]. A recent longitudinal study confirmed that incident dyslipidemia in police officers can be predicted by psychological distress [22].

It may be useful to employ two complementary models of job stress, DCS and ERI, to determine the typical conditions of police work [23]. In fact, the DCS model was originally developed to investigate typical male blue-collar working conditions, and is therefore sometimes considered to best assess aspects of task-oriented work, while emotional demands such related to confronting violent behavior, for example, are better measured by the ERI model [24].

The purpose of this study was to evaluate the association between stress and metabolic syndrome in a population of healthy individuals—the police officers of a rapid response unit. We chose a rapid response unit because these workers perform relatively few administrative tasks but are routinely engaged in maintaining law and order during demonstrations, meetings, sports events, natural calamities, riots, migration, and other unpredictable and sudden events. Our hypothesis was that, within this cohort, workers exposed to higher occupational stress have a higher incidence of metabolic syndrome or some of its components (hypertension, dyslipidemia, obesity, dyslipidemia).

## Methods

### Subjects and ethics statement

The study population was composed of police officers from the “VI Reparto Mobile” of Genoa. Participants were informed of the voluntary and confidential nature of the study through an information leaflet, and the study plan was approved by the Ethics Committee of the Università Cattolica del Sacro Cuore of Rome. Participants provided their written informed consent to participate in this study by signing a form in their own personal health file. Written informed consent for access to health records and linkage was obtained in compliance with indications in the literature [25] and the Italian Law on Privacy [26]. The study was conducted according to the ethical standards of the Declaration of Helsinki. All police officers of the “VI Reparto Mobile” were enrolled. Questionnaires were self-completed in a special room at police headquarters after medical examination and with no time limit on the morning of a rest day. Complete confidentiality was guaranteed, and a contact telephone number was provided for help in completing the questionnaire.

We began data collection in January 2009. Questionnaires for self-assessment of job stress were completed three times (January, April and July) in 2009, in 2011 and 2013. Blood tests were performed in January 2009 and in January 2014.

292 out of 294 workers participated in 2009; two workers of female gender were excluded in order to avoid gender bias. 234 police officers completed all the examinations. The main reason for abandoning the cohort was transfer to other police units (41 persons, 73.2%); 12 officers retired (21.4%), 3 deceased (5.4%)

In addition to the self-reported assessments, male subjects also provided biologic and physiologic data. The detailed information about their sociodemographic and work characteristics, including age, education, marital status, presence of offspring, type of housing, military rank, work experience, smoking habit, as well as their past medical history, was recorded through interview-based medical examinations. The risk factors for MetS were actively sought. All the members of the cohort did regular physical exercise. In fact, they performed mandatory physical-technical-tactical activities (8 hours per month), in addition to gym exercises in the barracks, jogging, running, cycling, CrossFit, and other physical activities for 9–12 hours a week. The officers ate in a canteen where the same menus, based on a correct nutritional intake, were available for all the staff. Work shifts, which often require overtime, were rotated but there were no night shifts. The presence of sleep problems was screened through questionnaires on sleep quantity/quality and excessive daytime sleepiness, plus medical examination, according to a validated protocol [27]. Socio-demographic parameters, smoking habit and sleep problems were coded as binary variables. Subject height and weight were recorded, and waist circumference was obtained by measuring at the narrowest point between the lower costal (tenth rib) and the iliac crest. BMI was calculated as weight (kg) divided by height squared (m^2^). Blood pressure was measured using a sphygmomanometer in a sitting position following a ten-minute rest. Venous fasting blood samples were collected in plain tubes and centrifuged at 3,000 rpm for 10 min at room temperature, and serum samples were frozen at -20°C until assayed. Fasting blood glucose (FBG) and blood lipid profile (total cholesterol, high-density lipoprotein, triglycerides, and low-density lipoprotein) were determined using an enzymatic method kit. All blood samples were analyzed at the S. Martino university hospital laboratory of Genoa.

Components of the metabolic syndrome were defined according to the International Diabetes Federation (IDF) guide [28], and the National Cholesterol Education Program Expert Panel on Detection Evaluation and Treatment of High Cholesterol in Adults (NCEP/ATPIII) [29], as follows: central obesity was defined as BMI>30 kg/m^2^, or a waist circumference of >85 cm in men. Hypertriglyceridemia was defined as a serum triglyceride level >150 mg/dL (1.7 mmol/L). Low high-density lipoprotein (HDL) cholesterolemia was defined as a serum HDL-cholesterol level of <40 mg/dL (1.03 mmol/L). High blood pressure was defined as a systolic blood pressure of >130 mmHg and/or a diastolic blood pressure of >85 mmHg. Blood pressure was measured at every medical examination in clinostatic and orthostatic position, at the same time in the morning to avoid the circadian effect [30–33]. High fasting glucose was defined by a plasma glucose level of >100 mg/dL (5.6 mmol/L).

### Occupational stress

Two standardized questionnaires were utilized for occupational stress assessment: the DCS questionnaire based on Karasek’s model and the effort-reward imbalance questionnaire (ERI) based on Siegrist’s ERI model. The Italian versions of the two questionnaires have been shown to be reliable and valid [34].

The 17-item DCS questionnaire can be divided into three scales: psychological demand (five questions), job control (six questions) and workplace social support (six questions). Participants answered according to the frequency of their feelings on a 4-point Likert scale ranging from 1 (never) to 4 (always). The value of the demand-control ratio (D/C ratio) was derived to evaluate the balance between psychological demand and job control. The D/C ratio formula was: demand/control × correction factor. The correction factor was introduced to correct the difference in the number of items on the two scales. A D/C ratio greater than 1 indicated job strain with high job demand and low job control. In this study, Cronbach’s α values for job demand and job control were 0.71 and 0.65, i.e. close to those found in the literature [35–39], indicating reasonably acceptable internal consistency [40].

The ERI questionnaire includes six items for job effort and eleven items for job reward. Items were scored on a Likert scale ranging from 1 (no) to 5 (yes, and I feel very distressed) for effort, and from 1 (I agree) to 5 (I don’t agree, and I feel very distressed) for reward. The value of the effort-reward ratio (E/R ratio) was calculated to evaluate the balance between job effort and reward. The E/R ratio formula was: effort/reward × correction factor. The correction factor corrects the difference in the number of items on the two scales. A score for the E/R ratio greater than 1 indicates a high-risk imbalance with high effort and low reward. In this study, Cronbach’s α values for job effort and job reward were 0.82 and 0.89, respectively, showing high internal consistency. The questionnaire also contains an over-commitment measurement that describes strong commitment toward work in addition to the difficulty of temporarily relaxing from work-related thoughts and activities. The over-commitment scale was composed of 6 items with a 4-point Likert scale; in the present study, Cronbach’s α was 0.79.

Two methods were used to quantify the average stress level experienced by each subject during the observation period. Firstly, we considered the stress levels within the population. The results of each of the two tests (D/C ratio and E/R ratio) were categorized into quartiles. The quartiles of the five measurements made between 2009 and 2013 were added and the resulting value was again categorized into quartiles. In this way, officers whose stress scores were lower than average in all measurements were placed in the first quartile, while officers with the highest stress levels were put in the fourth quartile. Secondly, we considered individual levels of stress. We calculated the mean value for the D/C weighted ratio and E/R weighted ratio of each police officer, and identified those who had job strain (D/C>1) or effort/reward imbalance (E/R>1) for the entire period (“high stress” persons). We also considered the average value of each of the six stress-related variables (demand, control, support in the DCS model; effort, reward, over-commitment in the ERI model) and studied the association(s) of these variables with output.

### Statistical analyses

First of all, we used one-way analysis of variance (Anova) to analyze differences in MetS components between groups defined by stress levels, and the Bonferroni post-hoc comparison of group means. In this way we were able to ascertain whether, within the population, those who perceived a higher level of occupational stress also had a higher frequency of metabolic disorders.

Secondly, we examined whether working with a high level of stress increased the risk of metabolic disorders. Univariate logistic regression analysis was used to study the association between job strain or effort/reward imbalance (D/C ratio >1 or E/R ratio >1) and the presence of metabolic syndrome. Multivariate methods were used to check for confounding factors. In the first model socio-demographic variables (age, rank, education, geographic origin, marital status, housing, offspring) were inserted as predictors; in the second model, in addition to the above variables, also lifestyle factors (smoking habit, sleep habits) were included.

Finally, we determined which of the stress variables was best correlated with the incidence of MetS, once more by univariate and multivariate logistic regression analyzes, using the average value of each of the six stress variables (demand, control, support, effort, reward, over-commitment) measured over the period as the predictor. Independent variables were carried forward to multivariable binary logistic regression for adjustment.

Analyses were performed using the Statistical Package for Social Science (IBM/ SPSS) for Windows (rel. 20.0).

## Results

At baseline, the mean age of the sample was 35.4+7.5 years. The average length of work experience in the police force was 14.0+7.9 years. More than half of the policemen (150, 51.7%) were officers, while the remainder were higher ranking (special officer, superintendent, inspector, technician) and had a slightly higher salary than police officers. All of them performed the same tasks, regardless of rank. According to the Italian educational system, 75.2% of the participants had an average or high level of education (high school diploma, 13 years of education; post-secondary school qualification, at least 16 years of education), while the remaining 24.8% had a low level of education (junior high school diploma, less than 10 years of education). Fifty percent of the participants was born in Northern Italy, the remaining in Southern Italy. 56.2% were quartered in barracks. Most of the officers (62.8%) were single or separated, while 37.2% were married, and 36.6% had at least one child. At baseline, the number of cases (prevalence) of obesity, high blood pressure, hypertriglyceridemia, low HDL-cholesterol, and high fasting glucose were 110 (37.9), 36 (12.4%), 34 (11.7%), 27 (9.3%), and 7 (2.4%), respectively. 14 persons out of 290 (4.8%) had MetS at baseline. The characteristics of the officers that completed the follow-up are reported in Table 1. We failed to observe any significant difference in personal and socio-economic characteristics, prevalence of MetS components and stress levels at baseline between those who completed the study and those who did not do so.

**Table 1:** Characteristics of the studied population at the end of the follow-up.

Social-demographic & lifestyle variables	
Gender, male N (%)	234 (100%)
Age, years (mean + s.d.)	41.0 ± 7.4
Working experience, years (mean + s.d.)	19.6 ± 7.8
Rank, higher than officer, N (%)	126 (53.8%)
Education level, high, N (%)	170 (72.6%)
Origin, Northern Italy, N (%)	120 (51.3%)
Marital status, married, N (%)	93 (39.7%)
Housing, barracks, N (%)	120 (51.3%)
Offspring, presence, N (%)	94 (40.2%)
Smokers, N (%)	66 (28.2%)
Sleep disorder, N (%)	77 (32.9%)
Job stress variables	
Demand (range 5–20) (mean + s.d.)	13.7±1.9
Control (range 6–24) (mean + s.d.)	12.7±2.5
Support (range 6–24) (mean + s.d.)	18.3±2.8
Effort (range 6–30) (mean + s.d.)	15.9±2.9
Reward (range 11–55) (mean + s.d.)	40.7±5.6
Overcommitment (range 6–24) (mean + s.d.)	7.1±2.1
Job strain (D/C >1) N (%)	207 (88.5%)
Effort/Reward imbalance (E/R>1) N (%)	29 (12.4%)
Incident cases	
Abdominal obesity (N, incidence ‰)	112 (140‰)
Hypertension (N, ‰)	41 (40.6‰)
Hypertriglyceridemia (N, ‰)	8 (8‰)
Low HDL-cholesterol (N, ‰)	11(11.6‰)
Diabetes (N, ‰)	3 (2.6‰)
Metabolic syndrome (N, ‰)	27(24.5‰)

During the 5 years of observation, of the subjects who completed the follow-up 207 police officers (88.5%) reported a mean D/C ratio>1 (i.e.: job strain), and 29 (12.4%) reported a mean E/R ratio>1 (i.e.: effort/reward imbalance). At the end of the observation period, the overall number of cases (prevalence) of obesity, high blood pressure, hypertriglyceridemia, low HDL-cholesterol, and high fasting glucose were 186 (79.5%, prevalently abdominal obesity), 73 (31.2%), 42 (17.9%), 37 (15.8%), and 10 (4.3%), respectively. In five years, the number of people with values exceeding the norm increased by 20–30% for each of the MetS components. New cases (incidence) of obesity, hypertension, hypertriglyceridemia, low HDL-cholesterol, and high fasting glucose were 112 (140.0 per thousand persons/year), 41 (40.6‰), 8 (8.0‰), 11 (10.6‰) and 3 (2.6‰), respectively. If compared to baseline, total cholesterol, triglyceride, and blood glucose mean levels had increased by 7.1+14.0, 5.5+14.3, and 0.4+2.3, while HDL-cholesterol had decreased on average by 1.5+3.4. All differences were highly significant (paired-samples Student’s t test *p*<0.001). The presence of three or more of the metabolic components, i.e. a diagnosis of MetS, was reported in 41 persons (17.5%). 27 new cases of MetS were observed among the 220 police officers who were previously unaffected (incidence rate 24.5 per thousand persons per year).

One-way Anova showed that between groups of workers characterized by different levels of stress. blood lipids values differed significantly, and post-hoc comparisons with the Bonferroni test confirmed that police officers with persistently lower levels of stress (Group 1, first quartile) had significantly lower mean levels of triglycerides and total cholesterol, and higher levels of HDL-cholesterol than their colleagues in the highest quartile of job strain (Table 2).

**Table 2:** Difference between groups exposed to different levels of work stress.

MetS component	Stress level				ANOVA F	*p*
	Lowest quartile N = 76	2^nd^ quartile N = 81	3^rd^ quartile N = 32	Highest quartile N = 45		
BMI	26.41+3.83	26.90+2.96	27.82+3.56	27.24+3.63	1.421	n.s.
Abdominal circumference	93.78+10.84	94.84+9.17	95.47+12.52	94.17+10.29	0.263	n.s.
Triglycerides	96.78+45.23*	111.11+41.63	116.69+44.80	131.13+50.43*	5.698	0.001
Total cholesterol	187.20+36.76*	196.53+31.50*	201.94+27.95	217.00+31.52*	7.949	0.001
HDL-cholesterol	50.25+8.43*	47.88+10.07	43.50+8.61*	46.07+6.87	5.080	0.002
Systolic pressure	132.43+23.24	130.00+20.21	125.78+15.51	126.66+12.29	1.343	n.s.
Diastolic pressure	78.02+9.28	78.07+9.05	76.41+8.73	77.09+7.25	0.392	n.s.
Blood glucose	81.66+10.11	82.86+9.41	83.34+10.30	82.33+8.35	0.392	n.s.

*Bonferroni post-hoc test significant at p<0.05

n.s.: not significant

Univariate logistic regression showed that job strain or effort/reward imbalance (D/C ratio or E/R ratio>1) significantly predicted the onset of metabolic syndrome. Police officers with stress ratio >1 during the observation period had a greater risk of developing metabolic syndrome (OR = 3.29; CI95% = 1.44–7.54) than colleagues with stress levels below the limit. This association was confirmed (OR = 2.68; CI95% = 1.07–6.70) after taking into account the socio-demographic (age, rank, education, geographic origin, marital status, housing, and presence of offspring) and lifestyle confounding factors (smoking habit and sleep habits) (Table 3). Work-related stress was significantly associated with incident cases of hypertriglyceridemia; the increase in the odds ratio remained significant even after correction (OR = 7.9, CI95% = 1.3–48.0). Distressed police officers also showed a non-significant increased risk of having high glucose and low HDL-cholesterol at follow-up (Table 3).

**Table 3:** Odds ratios and 95% confidence intervals of high stress level on incident cases of metabolic syndrome and its components.

	Unadjusted			Adjusted, Model 1*			Adjusted, Model 2**		
Output	OR	95% CI	*p*	OR	95% CI	*p*	OR	95% CI	*p*
Incident MetS	3.29	1.44–7.54	<0.01	3.50	1.48–8.26	<0.01	2.68	1.08–6.70	<0.05
Abdominal obesity	0.56	0.32–1.01	n.s.	0.59	0.33–1.04	n.s.	0.64	0.35–1.17	n.s.
Hypertension	0.58	0.29–1.17	n.s.	0.59	0.29–1.22	n.s.	0.67	0.31–1.44	n.s.
Hypertriglyceridemia	12.56	2.91–54.32	<0.01	14.63	2.83–75.77	<0.01	7.86	1.29–48.04	<0.05
Low HDL-cholesterol	2.11	0.63–7.12	n.s.	2.03	0.58–7.11	n.s.	1.57	0.40–6.25	n.s.
Diabetes	3.68	0.41–32.95	n.s.	6.36	0.56–72.45	n.s.	7.95	0.64–98.30	n.s.

*corrected for age, rank level, education, geographic origin, marital status, housing, and presence of offspring

** also corrected for lifestyle (smoking habit, sleep habits)

On analyzing each of the work-related stress variables separately, we observed that the average values of Demand and Effort were significant predictors of MetS (Table 4). The association was confirmed even after correction for confounders.

**Table 4:** Association of stress variables with incident cases of metabolic syndrome.

	Unadjusted			Adjusted, Model 1*			Adjusted, Model 2**		
Stress variable	OR	95% CI	*p*	OR	95% CI	*p*	OR	95% CI	*p*
Demand	1.41	1.14–1.74	<0.01	1.43	1.15–1.78	<0.01	1.36	1.08–1.70	<0.01
Control	0.93	0.79–1.08	n.s.	0.90	0.76–1.06	n.s.	0.93	0.78–1.10	n.s.
Support	0.93	0.81–1.06	n.s.	0.92	0.81–1.05	n.s.	0.94	0.82–1.07	n.s.
Effort	1.18	1.03–1.33	<0.05	1.21	1.05–1.39	<0.01	1.16	1.01–1.35	<0.05
Reward	0.97	0.91–1.03	n.s.	0.97	0.90–1.03	n.s.	0.99	0.92–1.07	n.s.
Overcommitment	1.15	1.01–1.33	0.05	1.16	1.01–1.35	<0.05	1.14	0.98–1.33	n.s.

*corrected for age, rank level, education, geographic origin, marital status, housing, and presence of offspring

** also corrected for lifestyle (smoking habit, sleep habits)

## Discussion and Conclusions

Our study, conducted on a small group of highly selected police workers with a much better health level than the general population [41, 42], confirmed that work-related stress may induce metabolic syndrome.

Many previous studies have indicated that life stress leads to MetS [43], and that stress may also arise from occupational problems [44]. However, separating work and life stress is not easy on account of the difficulty of observing a population in which occupational stressors are markedly dominant. In our study, the continuous exposure of police officers to risks associated with the maintenance of law made it possible to neglect stressful life events. Occupational stress was associated with MetS in a number of cross-sectional observations, using the ERI model [45, 46], or other validated questionnaires [47, 8], as well as in longitudinal studies, which adopted measures of self-perceived stress [48], organizational justice [49] or job strain according to Karasek’s model [11]. Distress, measured at the baseline, was also associated with an increased risk of MetS at follow-up [50]. All of these studies support the hypothesis that prolonged occupational stress can increase the risk of MetS. However, no study has succeeded in continuously measuring stress levels throughout the period of observation. Authors assume that stress levels remain constant for years, but there is no evidence of this. On the contrary, longitudinal surveys show that self-perceived levels of occupational stress generally tend to decrease with an increase in the years of occupation in the same company [51]. For this reason we believe it is important to continuously monitor the level of stress in occupational studies.

In literature there are several studies on stress and MetS in the general population. Meta-analysis and systematic reviews of cross-sectional and baseline longitudinal studies in adults confirm that MetS is highly prevalent in people exposed to such a degree of both chronic [12] and acute stress that it induces post-traumatic stress disorder [52, 53]. In our study the use of two complementary measures of self-perceived stress (DCS and ERI questionnaires) enabled us to study both major traumas and a succession of minor psychological trauma in everyday work.

In this study we observed that among the measures of stress, Demand is the most important in bringing about MetS. Effort is also associated with MetS in unadjusted and adjusted models. These variables express the weight of work in the two concurrent models of stress. Since our research focused on a very specific type of job, we do not know if this finding can be generalized to all workers. However, our results indicate that, in this police unit, stress management should aim to reduce the load rather than to increase job control or rewards. Among MetS components in this cohort, blood lipids seemed to have the strongest association with job stress. Distressed officers had a marked increased risk of hypertriglyceridemia. Stress increased the odds for high glucose, and low HDL-cholesterol, although not significantly. Caution should be taken in interpreting these findings, given the limited number of observations. Future studies, conducted on larger cohorts or for longer periods, will clarify whether job stress is associated with all MetS components. However, the association we found between job stress and lipid change is in accordance with the literature. The prospective population study of Puustinen et al. [50] reported that psychological distress is a significant predictor of HDL-cholesterol levels and causes a non significant increase in triglyceride levels. Work stress, measured with the demand/control model, was associated with an increase in total cholesterol level [54]. From the Whitehall II study cohort, data on justice at work were associated with the development of reduced HDL-cholesterol and elevated triglycerides in men but not in women [49]. Blood lipid levels have been used as a measure of subclinical atherosclerosis and have been shown to predict coronary heart disease events [55, 56].

Our findings are in agreement with the literature on police officers. Cross-sectional data from the Buffalo Cardio-Metabolic Occupational Police Stress (BCOPS) Study (2004–2009) recently showed that police officers have high prevalence of MetS (25.7%) and reduced cortisol response to a high-protein meal challenge, that may be associated with MetS [57]. A high prevalence of MetS and an association with work stress were also found in two cross-sectional studies of Indian police officers [20, 58].

As regards the causes of MetS in police, one of the most important is psychological trauma connected with facing or witnessing violence. Abnormal serum lipid profile has been found in Brazilian police officers with post-traumatic stress disorder [59]. Depression, one of the major consequences of stress, is associated with MetS in police officers [60]. However, many other elements such as dietary factors [61], lifestyle [62], atypical work hours [63], reduced sleep duration [64, 65], lack of physical activity [66], or a combination of all these factors [67] play a causative role in MetS in police. In our cohort, we were able to rule out the role of dietary factors, shift and night work, lack of physical activity, and depression or other common mental disorders; however, when we checked the effect of smoking and sleep habits on MetS; the latter proved to be a very important factor. A study of the relationship between sleep habits, work stress, and MetS would be useful to clarify their mutual effects.

All the aforementioned studies on police are cross-sectional. We found only one follow-up study that reported an increased incidence of dyslipidemia in Chinese police officers in charge of traffic control [22]. Further longitudinal studies of the type we conducted could provide useful evidence for planning intervention on work-related stress, MetS and associated health risks among police officers.

With regard to possible underlying mechanisms of MetS, there is evidence that the association between work stress and MetS is mediated through indirect effects on health behavior as well as direct effects on neuroendocrine stress pathways [48]. Cross-sectional [68, 69] and longitudinal studies [70, 71] have shown that occupational stress is often associated with unhealthy behavior and obesity. Chronic stress increases vulnerability to diet-related abdominal fat, and oxidative stress [72], which may in turn induce insulin resistance, dyslipidemia, and impaired glucose tolerance [73].

The main strength of our study was the repeated measurement of stress during the whole period of observation. To the best of our knowledge, our study is the only one in which the subjects classified as "high stress" remained thus for the entire duration of the observations. Though stress was high in the cohort, it did not result in an early withdrawal, as shown by the fact that the mean stress level at the end of the observation period is comparable to the baseline level. Workers participated in the study with great diligence, and the loss of cases was almost exclusively due to transfer to other units. This occurred because the policemen acknowledged that stress is a major risk in their occupation. Excessive stress in a police officer responsible for maintaining law and order is not only a risk for his/her own health, but also for the health and safety of others.

Another strong point was the choice of questionnaires used for measuring stress (those most widely adopted in the occupational field [12]), since this allowed a comparison with other groups of workers [74].A limiting factor in our study regards the health status of rapid response police officers who are a highly selected corps. Previous studies showed that this sample had a better level of mental health [41] and a lower rate of absence due to illness than the general population of workers [42]. This is to be expected as flying squad officers who suffer from moderate/severe illness are transferred to administrative posts. Studies on MetS confirm that metabolic syndrome and its components occur more frequently in the general population than was observed in our sample. The Italian Longitudinal Study on Aging (ILSA) reported a MetS prevalence of 25.9% [75]. Another study, that used IDF criteria, estimated a 28.0% prevalence of undiagnosed MetS in the general population [76]. A stratified sample of the Veneto region (North East Italy) reported a prevalence of 8.0% in individuals between the age of 30–39 years, that increased to 29.8% in in subjects aged 60–69 years [77]. A study conducted in the Marches region of Italy found a MetS prevalence of 11.5% in the 36 to 42 age group and of 22.5% in subjects between the age of 43 and 60 years [78]. It is estimated that in Europe around 20% of adults have a metabolic syndrome [79], with higher prevalence in selected groups. The age-standardized percentage of obese subjects with MetS ranged from 43% to 78% in men in different European cohorts, with elevated blood pressure the most frequently occurring factor contributing to the prevalence of MetS [80]. The prevalence in population-based surveys for male subjects is 20% in Oman [81], 26% in China [81], 27% in Turkey [81], 29.6% in Brazil [82], 25% in adults in the U.S., with higher rates among racial/ethnic minority groups [83]. A recent meta-analysis showed that the pooled MetS prevalence is 38.7% in individuals with post-traumatic stress disorder (PTSD) [84]. The third National Health and Nutrition Examination Survey (NHANES III) recorded a MetS prevalence in the total population of 21.8% [85]. Despite the increase observed during our study, the prevalence of MetS at follow-up (17.5%) in our sample was less than the aforementioned prevalence data for the general Italian population and other industrialized countries. Nevertheless, even in this healthy population, occupational stress played a significant role in the development of MetS and its components. In our view, this reinforces our findings on the association between stress and metabolic syndrome.

Another limitation of our study is the scant number of observations and the lack of a control group, although both these conditions are very common in occupational medicine. On the positive side, our sample was very homogeneous, because all the participants were engaged daily in maintaining law and order, whereas studies on police often include workers who perform mainly administrative tasks and therefore have a risk profile that differs considerably from those who maintain order.

This study adds further support to the idea that stress should be prevented because it causes diseases. Every effort must be made to avoid minimizing the problem. Prevention needs to be applied to healthy individuals in order to avoid distress that may result in disease.

The well-being of workers engaged in law enforcement is a guarantee of safety for the community. For this reason we must focus on the prevention of stress in police officers and combat the stigma associated with distress and disease. Our findings should encourage researchers to carry out more prospective studies. Programs designed to reduce work stress and improve lifestyles could provide significant advantages for police officers in terms of health and productivity.

## Supporting Information

S1 Data Data, to be deposited on Dryad repository, as soon as the work will be accepted. (XLSX)

## References

[1] Mottillo S, Filion KB, Genest J, Joseph L, Pilote L, Poirier P, et al The metabolic syndrome and cardiovascular risk: a systematic review and meta-analysis. J Am Coll Cardiol 2010 56 1113–1132. (10.1016/j.jacc.2010.05.034) 20863953

[2] Esposito K, Chiodini P, Colao A, Lenzi A, Giugliano D. Metabolic syndrome and risk of cancer: a systematic review and meta-analysis. Diabetes Care. 2012;35(11):2402–11. 10.2337/dc12-0336 23093685 PMC3476894

[3] Bhandari R, Kelley GA, Hartley TA, Rockett IR. Metabolic syndrome is associated with increased breast cancer risk: a systematic review with meta-analysis. Int J Breast Cancer. 2014;2014:189384 10.1155/2014/189384 25653879 PMC4295135

[4] Siervo M, Harrison SL, Jagger C, Robinson L, Stephan BC. Metabolic syndrome and longitudinal changes in cognitive function: a systematic review and meta-analysis. J Alzheimers Dis. 2014;41(1):151–61. 10.3233/JAD-132279 24577475

[5] Magnavita N, Fileni A. Work stress and metabolic syndrome in radiologists: first evidence. Radiol Med. 2014;119(2):142–8. 10.1007/s11547-013-0329-0 24297580

[6] Magnavita N. Work-related psychological injury is associated with metabolic syndrome components in apparently healthy workers. PLoS One, 2015; 10(6):e0130944 10.1371/journal.pone.0130944 26086387 PMC4473100

[7] Kang MG, Koh SB, Cha BS, Park JK, Woo JM, Chang SJ. Association between job stress on heart rate variability and metabolic syndrome in shipyard male workers. Yonsei Med J. 2004;45(5):838–46. 15515194 10.3349/ymj.2004.45.5.838

[8] Kawada T. Relationship between components of the metabolic syndrome and job strain using a brief job stress questionnaire (BJSQ). Int Arch Occup Environ Health. 2013;86(6):725–6. 10.1007/s00420-013-0870-0 23584233

[9] Djindjic N, Jovanovic J, Djindjic B, Jovanovic M, Jovanovic JJ. Associations between the occupational stress index and hypertension, type 2 diabetes mellitus, and lipid disorders in middle-aged men and women. Ann Occup Hyg. 2012;56(9):1051–62. 10.1093/annhyg/mes059 Epub 2012 Sep 17. 22986427

[10] Chandola T, Brunner E, Marmot M. Chronic stress at work and the metabolic syndrome: prospective study. BMJ. 2006;332:521–525. 16428252 10.1136/bmj.38693.435301.80 PMC1388129

[11] Edwards EM, Stuver SO, Heeren TC, Fredman L. Job strain and incident metabolic syndrome over 5 years of follow-up: the coronary artery risk development in young adults study. J Occup Environ Med 2012 54 1447–1452. (10.1097/JOM.0b013e3182783f27) 23171915

[12] Bergmann N, Gyntelberg F, Faber J. The appraisal of chronic stress and the development of the metabolic syndrome: a systematic review of prospective cohort studies. Endocr Connect. 2014;3(2):R55–80. 10.1530/EC-14-0031 24743684 PMC4025474

[13] Karasek RA. Job demands. job decision latitude. and mental strain. Implication for job redesign. Adm Sci Q 1979; 24: 285–308

[14] Siegrist J. Adverse health effects of high-effort/low-reward conditions. J Occup Health Psychol 1996; 1:27–41 9547031 10.1037//1076-8998.1.1.27

[15] Kivimäki M, Nyberg ST, Batty GD, Fransson EI, Heikkilä K, Alfredsson L, et al Job strain as a risk factor for coronary heart disease: a collaborative meta-analysis of individual participant data. Lancet 2012, 380(9852):1491–1497. 10.1016/S0140-6736(12)60994-5 22981903 PMC3486012

[16] Nieuwenhuijsen K, Bruinvels D, Frings-Dresen M. Psychosocial work environment and stress-related disorders, a systematic review. Occup Med 2010, 60(4):277–286. 10.1093/occmed/kqq081 20511268

[17] Janczura M, Bochenek G, Nowobilski R, Dropinski J, Kotula-Horowitz K, Laskowicz B, et al The relationship of metabolic syndrome with stress, coronary heart disease and pulmonary function—an occupational cohort-based study. PLoS One. 2015;10(8):e0133750 10.1371/journal.pone.0133750 26274823 PMC4537246

[18] Joseph PN, Violanti JM, Donahue R, Andrew ME, Trevisan M, Burchfiel CM, et al Police work and subclinical atherosclerosis. J Occup Environ Med. 2009;51 (6):700–707. 19530342 10.1097/jom.0b013e3181a02252

[19] Kumar P, Mallik D, Mukhopadhyay DK, Sinhababu A, Mahapatra BS, Chakrabarti P. Prevalence of diabetes mellitus, impaired fasting glucose, impaired glucose tolerance, and its correlates among police personnel in Bankura District of West Bengal. Indian J Public Health. 2013;57:24–28. 10.4103/0019-557X.111364 23649139

[20] Walvekar SS, Ambekar JG, Devaranavadagi BB. Study on serum cortisol and perceived stress scale in the police constables. J Clin Diagn Res. 2015;9(2):BC10–4. 10.7860/JCDR/2015/12015.5576 25859444 PMC4378726

[21] Hartley TA, Burchfiel CM, Fekedulegn D, Andrew ME, Knox SS, Violanti M. Associations between police officer stress and the metabolic syndrome. Int J Emerg Ment Health. 2011;13:243–256 22900458 PMC4734368

[22] Chen X, Leng L, Yu H, Yang XL, Dong GH, Yue S, et al Psychological distress and dyslipidemia in Chinese police officers: a 4-year follow-up study in Tianjin, China. J Occup Environ Med. 2015;57(4):400–5. 10.1097/JOM.0000000000000372 25629802

[23] Magnavita N, Garbarino S, Siegrist J. Stress in police: assessment methods. G It Med Lav Ergon 2014; 36 (4): 400–404 25558743

[24] Söderberg M, Rosengren A, Hillström J, Lissner L, Torén K. A cross-sectional study of the relationship between job demand-control, effort-reward imbalance and cardiovascular heart disease risk factors. BMC Public Health 2012, 12:1102 10.1186/1471-2458-12-1102 23259757 PMC3541343

[25] da Silva ME, Coeli CM, Ventura M, Palacios M, Magnanini MM, Camargo TM, et al Informed consent for record linkage: a systematic review. J Med Ethics. 2012;38(10):639–42. 22403083 10.1136/medethics-2011-100208

[26] Legislative Decree 196, 30/6/2003 “Codice in materia di protezione dei dati personali”. Available at: http://garanteprivacy.it/web/guest/home/docweb/-/docweb-display/docweb/1311248

[27] Garbarino S, Magnavita N. Obstructive sleep apnea syndrome (OSAS), metabolic syndrome and mental health in small enterprise workers. Feasibility of an action for health. PLoS One. 2014; 9(5): e97188 10.1371/journal.pone.0097188 24810290 PMC4014618

[28] International Diabetes Foundation IDF. 2006. The IDF consensus worldwide definition of the metabolic syndrome. Available: http://www.idf.org/webdata/docs/IDF_Meta_def_final.pdf

[29] National Cholesterol Education Program: Executive summary of the third report of the National Cholesterol Education Program (NCEP) Expert Panel on Detection, Evaluation, and Treatment of High Blood Cholesterol in Adults (Adult Treatment Panel III). JAMA 2001; 285: 2486–2497 11368702 10.1001/jama.285.19.2486

[30] Tsimakouridze EV, Alibhai FJ, Martino TA. Therapeutic applications of circadian rhythms for the cardiovascular system. Front. Pharmacol. 2015; 6:77 10.3389/fphar.2015.00077 25941487 PMC4400861

[31] Hermida RC, Ayala DE, Mojón A, Fernández JR. Influence of circadian time of hypertension treatment on cardiovascular risk: results of the MAPEC study. Chronobiol Int. 2010;27(8):1629–51. 10.3109/07420528.2010.510230 20854139

[32] Hermida RC. Ambulatory blood pressure monitoring in the prediction of cardiovascular events and effects of chronotherapy: rationale and design of the MAPEC study. Chronobiol Int. 2007;24(4):749–75. 17701685 10.1080/07420520701535837

[33] Dashti HS, Aslibekyan S, Scheer FA, Smith CE, Lamon-Fava S, Jacques P, et al Clock Genes Explain a Large Proportion of Phenotypic Variance in Systolic Blood Pressure and This Control Is Not Modified by Environmental Temperature. Am J Hypertens. 2015 6 4. pii: hpv082. [Epub ahead of print] 10.1093/ajh/hpv082 PMC5863877 26045533

[34] Magnavita N. Two tools for health surveillance of job stress: the Karasek Job Content Questionnaire and the Siegrist Effort Reward Imbalance Questionnaire. G Ital Med Lav Ergon 2007; 29 (3): 667–670 18409896

[35] Sale JE, Kerr MS. The psychometric properties of Karasek's demand and control scales within a single sector: data from a large teaching hospital. Int Arch Occup Environ Health. 2002;75(3):145–52. 11954981 10.1007/s004200100289

[36] Niedhammer I. Psychometric properties of the French version of the Karasek Job Content Questionnaire: a study of the scales of decision latitude, psychological demands, social support, and physical demands in the GAZEL cohort. Int Arch Occup Environ Health. 2002;75(3):129–44. 11954980 10.1007/s004200100270

[37] Griep RH, Rotenberg L, Vasconcellos AG, Landsbergis P, Comaru CM, Alves MG. The psychometric properties of demand-control and effort-reward imbalance scales among Brazilian nurses. Int Arch Occup Environ Health. 2009;82(10):1163–72. 10.1007/s00420-009-0460-3 19756699

[38] Sanne B, Torp S, Mykletun A, Dahl AA. The Swedish Demand-Control-Support Questionnaire (DCSQ): factor structure, item analyses, and internal consistency in a large population. Scand J Public Health. 2005;33(3):166–74. 16040456 10.1080/14034940410019217

[39] Fujishiro K, Landsbergis PA, Diez-Roux AV, Stukovsky KH, Shrager S, Baron S. Factorial invariance, scale reliability, and construct validity of the job control and job demands scales for immigrant workers: the multi-ethnic study of atherosclerosis. J Immigr Minor Health. 2011;13(3):533–40. 10.1007/s10903-010-9364-2 20582720 PMC4068014

[40] Pelfrene E, Vlerick P, Mak R, Smets Pd, Kornitzer M, Backer Gd. Scale reliability and validity of the Karasek ‘Job Demand–Contol–Support’ model in the Belstress study. Work Stress 2001;15:297–313.

[41] Garbarino S, Cuomo G, Chiorri C, Magnavita N. Association of work-related stress with mental health problems in a special police force. BMJOpen 2013;3(7). pii: e002791. 10.1136/bmjopen-2013-002791 PMC3717472 23872288

[42] Magnavita N, Garbarino S. Is absence related to work stress? A repeated cross-sectional study on a special police force. Am J Ind Med. 2013; 56(7):765–75. 10.1002/ajim.22155 23334868

[43] Steptoe A, Kivimäki M. Stress and cardiovascular disease: an update on current knowledge. Annu Rev Public Health. 2013;34:337–54. 10.1146/annurev-publhealth-031912-114452 23297662

[44] Pickering TG. Mental stress as a causal factor in the development of hypertension and cardiovascular disease. Curr Hypertens Rep. 2001;3:249–254. 11353576 10.1007/s11906-001-0047-1

[45] Almadi T, Cathers I, Chow CM. Associations among work-related stress, cortisol, inflammation, and metabolic syndrome. Psychophysiology. 2013;50(9):821–30. 10.1111/psyp.12069 23758414

[46] Schmidt B, Bosch JA, Jarczok MN, Herr RM, Loerbroks A, van Vianen AE, et al Effort-reward imbalance is associated with the metabolic syndrome—findings from the Mannheim Industrial Cohort Study (MICS). Int J Cardiol. 2015;178:24–8. 10.1016/j.ijcard.2014.10.115 25464211

[47] Yamamoto K1, Okazaki A, Ohmori S. The relationship between psychosocial stress, age, BMI, CRP, lifestyle, and the metabolic syndrome in apparently healthy subjects. J Physiol Anthropol. 2011;30(1):15–22. 21307616 10.2114/jpa2.30.15

[48] Chandola T, Britton A, Brunner E, Hemingway H, Malik M, Kumari M, et al Work stress and coronary heart disease: what are the mechanisms? Eur Heart J 2008; 29(5):640–648 10.1093/eurheartj/ehm584 18216031

[49] Gimeno D, Tabák AG, Ferrie JE, Shipley MJ, De Vogli R, Elovainio M, et al Justice at work and metabolic syndrome: the Whitehall II study. Occup Environ Med. 2010;67(4):256–62. 10.1136/oem.2009.047324 19819861 PMC3226946

[50] Puustinen PJ, Koponen H, Kautiainen H, Mantyselka P, Vanhala M. Psychological distress predicts the development of the metabolic syndrome: a prospective population-based study. Psychosom Med 2011; 73: 158–165. (10.1097/PSY.0b013e3182037315) 21148808

[51] Magnavita N. Workplace violence and occupational stress in health care workers: a chicken and egg situation—Results of a 6-year follow-up study. J Nurs Scholarsh 2014; 46:5, 366–376 10.1111/jnu.12088 24754800

[52] Bartoli F, Carrà G, Crocamo C, Carretta D, Clerici M. Metabolic syndrome in people suffering from posttraumatic stress disorder: a systematic review and meta-analysis. Metab Syndr Relat Disord. 2013;11(5):301–8. 10.1089/met.2013.0010 23758060

[53] Rosenbaum S, Stubbs B, Ward PB, Steel Z, Lederman O, Vancampfort D. The prevalence and risk of metabolic syndrome and its components among people with posttraumatic stress disorder: a systematic review and meta-analysis. Metabolism. 2015;64(8):926–933. 10.1016/j.metabol.2015.04.009 25982700

[54] Kivimaki M, Leino-Arjas P, Luukkonen R, Riihimaki H, Vahtera J, Kirjonen J. Work stress and risk of cardiovascular mortality: prospective cohort study of industrial employees. BMJ 2002 325 857 (10.1136/bmj.325.7369.857) 12386034 PMC129630

[55] Wilson MG, Hamilton B, Sandridge AL, Salah O, Chalabi H. Differences in markers of cardiovascular disease between professional football players of West-Asian and Black African descent. J Sci Med Sport. 2012;15:266–271 10.1016/j.jsams.2011.10.007 22169212

[56] Zhou L, Ding H, Zhang X, et al Genetic variants at newly identified lipid loci are associated with coronary heart disease in a Chinese Han population. PloS One. 2011;6:e27481 10.1371/journal.pone.0027481 22110658 PMC3215720

[57] Baughman P, Andrew ME, Burchfiel CM, Fekedulegn D, Hartley TA, Violanti JM, et al High-protein meal challenge reveals the association between the salivary cortisol response and metabolic syndrome in police officers. Am J Hum Biol. 2015 6 19 10.1002/ajhb.22748 PMC4684818 26088798

[58] Thayyil J, Jayakrishnan TT, Raja M, Cherumanalil JM. Metabolic syndrome and other cardiovascular risk factors among police officers. N Am J Med Sci. 2012;4:630–635. 10.4103/1947-2714.104313 23272304 PMC3530318

[59] Maia DB, Marmar CR, Mendlowicz MV, Metzler T, Nóbrega A, Peres MC, et al Abnormal serum lipid profile in Brazilian police officers with post-traumatic stress disorder. J Affect Disord. 2008;107(1–3):259–63. 17888517 10.1016/j.jad.2007.08.013 PMC3974924

[60] Hartley TA, Knox SS, Fekedulegn D, Barbosa-Leiker C, Violanti JM, Andrew ME, et al Association between depressive symptoms and metabolic syndrome in police officers: results from two cross-sectional studies. J Environ Public Health. 2012;2012:861219 10.1155/2012/861219 22315628 PMC3270419

[61] Wirth MD, Burch J, Shivappa N, Violanti JM, Burchfiel CM, Fekedulegn D, et al Association of a dietary inflammatory index with inflammatory indices and metabolic syndrome among police officers. J Occup Environ Med. 2014;56(9):986–9. 10.1097/JOM.0000000000000213 25046320 PMC4156884

[62] Braga Filho RT, D'Oliveira A Jr. Metabolic syndrome and military policemen's quality of life: an interdisciplinary comprehensive approach. Am J Mens Health. 2014;8(6):503–9. 10.1177/1557988314526750 24626602

[63] Violanti JM, Burchfiel CM, Hartley TA, Mnatsakanova A, Fekedulegn D, Andrew ME, et al Atypical work hours and metabolic syndrome among police officers. Arch Environ Occup Health. 2009;64(3):194–201. 10.1080/19338240903241259 19864222

[64] McCanlies EC, Slaven JE, Smith LM, Andrew ME, Charles LE, Burchfiel CM, et al Metabolic syndrome and sleep duration in police officers. Work. 2012;43(2):133–9. 22927620 10.3233/WOR-2012-1399

[65] Yoo H, Franke WD. Sleep habits, mental health, and the metabolic syndrome in law enforcement officers. J Occup Environ Med. 2013;55(1):99–103. 10.1097/JOM.0b013e31826e294c 23207742

[66] Yoo HL, Eisenmann JC, Franke WD. Independent and combined influence of physical activity and perceived stress on the metabolic syndrome in male law enforcement officers. J Occup Environ Med. 2009;51(1):46–53. 10.1097/JOM.0b013e31817f9e43 19136873

[67] Song FJ, Tang NJ, Li SX, Yu H, Chen X, Song GX. A matched nested case-control study on the risk factors of metabolic syndrome among male criminal policemen. Zhonghua Lao Dong Wei Sheng Zhi Ye Bing Za Zhi. 2013;31(11):834–8. 24370293

[68] Nomura K, Nakao M, Tsurugano S, Takeuchi T, Inoue M, Shinozaki Y, et al Job stress and healthy behavior among male Japanese office workers. Am J Ind Med. 2010;53(11):1128–34. 10.1002/ajim.20859 20957727

[69] Magnavita N, Fileni A, Magnavita G, Mammi F, Mirk P, Roccia K, et al Work stress in radiologists. A pilot study. Radiol Med. 2008;113(3):329–46. 10.1007/s11547-008-0259-4 18493771

[70] Allard KO, Thomsen JF, Mikkelsen S, Rugulies R, Mors O, Kaergaard A, et al Effects of psychosocial work factors on lifestyle changes: a cohort study. J Occup Environ Med 2011 53 1364–1371.(10.1097/JOM.0b013e3182363bda) 22104976

[71] Berset M, Semmer NK, Elfering A, Jacobshagen N, Meier LL. Does stress at work make you gain weight? A two-year longitudinal study. Scand J Work Environ Health 2011 37 45–53. (10.5271/sjweh.3089) 20835689

[72] Aschbacher K, Kornfeld S, Picard M, Puterman E, Havel PJ, Stanhope K, et al Chronic stress increases vulnerability to diet-related abdominal fat, oxidative stress, and metabolic risk. Psychoneuroendocrinology. 2014;46:14–22. 10.1016/j.psyneuen.2014.04.003 24882154 PMC4104274

[73] Tangvarasittichai S. Oxidative stress, insulin resistance, dyslipidemia and type 2 diabetes mellitus. World J Diabetes. 2015;6(3):456–80. 10.4239/wjd.v6.i3.456 25897356 PMC4398902

[74] Magnavita N, Garbarino S. Social psychiatry in the waiting room. What a physician can learn about occupational stress from workers waiting to be examined. Psychiatry J. 2013;2013:701872 10.1155/2013/701872 24286068 PMC3820074

[75] Maggi S, Noale M, Gallina P, Bianchi D, Marzari C, Limongi F. Metabolic syndrome, diabetes and cardiovascular disease in an elderly Caucasian cohort: the Italian Longitudinal Study on Aging. J Gerontol A Biol Sci Med Sci 2006; 61:505–10 16720749 10.1093/gerona/61.5.505

[76] Bo S, Ciccone G, Pearce N, Merletti F, Gentile L, Cassader M, et al Prevalence of undiagnosed metabolic syndrome in a population of adult asymptomatic subjects. Diabetes Res Clin Pract. 2007;75(3):362–5. 16930757 10.1016/j.diabres.2006.06.031

[77] Novelletto BF, Guzzinati S, Avogaro A. Prevalence of metabolic syndrome and its relationship with clinically prevalent cardiovascular disease in the Veneto region, northeastern Italy. Metab Syndr Relat Disord. 2012;10(1):56–62. 10.1089/met.2011.0065 22004284

[78] Copertaro A. Prevalence of metabolic syndrome among forestry department agents in the Marche Region (Italy) Epidemiol Prev 2009; 33 (6): 227–232 20418576

[79] Scheen AJ, Luyckx FH. Metabolic syndrome: definitions and epidemiological data. Rev Med Liege. 2003;58(7–8):479–84. 14579611

[80] van Vliet-Ostaptchouk JV, Nuotio ML, Slagter SN, Doiron D, Fischer K, Foco L, et al The prevalence of metabolic syndrome and metabolically healthy obesity in Europe: a collaborative analysis of ten large cohort studies. BMC Endocr Disord. 2014;14:9 10.1186/1472-6823-14-9 24484869 PMC3923238

[81] Beck-Nielsen H. (ed.), The Metabolic Syndrome, Springer-Verlag Wien 2013 10.1007/978-3-7091-1331-8_2

[82] de Carvalho Vidigal F, Bressan J, Babio N, Salas-Salvadó J. Prevalence of metabolic syndrome in Brazilian adults: a systematic review. BMC Public Health. 2013;13:1198 10.1186/1471-2458-13-1198 24350922 PMC3878341

[83] Falkner B, Cossrow ND. Prevalence of metabolic syndrome and obesity-associated hypertension in the racial ethnic minorities of the United States. Curr Hypertens Rep. 2014;16(7):449 10.1007/s11906-014-0449-5 24819559 PMC4083846

[84] Rosenbaum S, Stubbs B, Ward PB, Steel Z, Lederman O, Vancampfort D. The prevalence and risk of metabolic syndrome and its components among people with posttraumatic stress disorder: a systematic review and meta-analysis. Metabolism. 2015;64(8):926–33. 10.1016/j.metabol.2015.04.009 25982700

[85] Ford ES, Giles WH, Dietz WH. Prevalence of the metabolic syndrome among US adults: findings from the Third National Health and Nutrition Examination Survey. JAMA 2002; 287: 356–359. 11790215 10.1001/jama.287.3.356

